# Comparison between grip strength and grip strength divided by body weight in their relationship with metabolic syndrome and quality of life in the elderly

**DOI:** 10.1371/journal.pone.0222040

**Published:** 2019-09-06

**Authors:** Se-Woong Chun, Won Kim, Kyoung Hyo Choi

**Affiliations:** 1 Department of Rehabilitation Medicine, Gyeongsang National University Changwon Hospital, Changwon, Gyeongsangnam-do, South Korea; 2 Department of Rehabilitation Medicine, Asan Medical Center, University of Ulsan College of Medicine, Seoul, South Korea; Nagoya University, JAPAN

## Abstract

Strength measures should be normalized by body mass; however, the definition of sarcopenia includes only simple grip strength. Thus, we compared the relationship of grip strength and grip strength divided by body weight or body mass index to two major consequences of sarcopenia, namely metabolic syndrome and poor quality of life. Data from the participants (aged 60 years or older) of the Sixth Korea National Health and Nutrition Examination were analyzed. Metabolic syndrome was defined according to the Adult Treatment Panel III guidelines with some modifications appropriate for Koreans. Quality of life was assessed using the EuroQoL Five-dimension questionnaire. Multiple logistic regression models were used to evaluate the association of grip strength and grip strength divided by body weight with metabolic syndrome and quality of life. A total of 1273 men and 1436 women were included in the analyses. Grip strength was not related to metabolic syndrome, whereas grip strength divided by body weight and grip strength normalized by body mass index revealed a dense dose-response relationship. All measures showed a similar correlation with quality of life. Grip strength divided by body weight can be superior to simple grip strength and grip strength normalized by body mass index in representing the metabolic aspects of sarcopenia.

## Introduction

The skeletal muscle generates force with carbohydrates and fats as fuel [1], and enables physical activity with the help of the skeleton [2]. It also secretes various myokines that influence glucose and fatty acid metabolism [3]. Thus, the aging-associated physiologic deterioration in the mass and function of the skeletal muscle [4] has important consequences. It contributes to increased disabilities [5] and metabolic disorders [6], ultimately hampering the quality of life (QoL) [7] and increasing the all-cause mortality rates [8]. This is why disproportionate age-related skeletal muscle wasting, so-called sarcopenia, has generated great interest among clinicians and medical professionals in the past few decades.

The definition of sarcopenia includes three dimensions: muscle mass, muscle strength, and physical performance [9]. However, there is no current consensus on the specific criteria for each dimension. With respect to muscle mass, Kim et al. reviewed the indices of this variable derived by height, weight, and body mass index (BMI), and reported the changes in the prevalence of sarcopenia by applying these different criteria [10]. Similar analyses of muscle strength are warranted but none have been performed to date.

Major working groups on sarcopenia have adopted simple grip strength as a measure of muscle strength [11–13]. However, it has been proposed that strength adjusted by body size might be superior to simple strength in predicting either a diseased state [14] or mobility and QoL [15]. Many researchers have investigated the relationship of muscle strength to metabolic disorders [16,17] and QoL [15,18]. Some used raw muscle strength [17,18], whereas others adopted muscle strength normalized by body weight (bwt) [15,16]. This could be the reason why the results differ to some extent across earlier studies [19]. However, no studies to date have evaluated the effect of normalizing strength measures by body mass on the association between strength and metabolic syndrome or QoL.

The current study was conducted to outline age-related changes in grip strength, grip strength normalized by bwt, and grip strength normalized by BMI, and to compare these three variables in terms of their relationship to metabolic syndrome or QoL by using data from a nationwide Korean survey.

## Materials and methods

### Study population

The data used in this study were extracted from the Sixth Korea National Health and Nutrition Examination Surveys (KNHANES VI-1,2), acquired from January 2014 to December 2015. KNHANES is a nationwide, cross-sectional survey conducted by the Korea Centers for Disease Control and Prevention [20]. A stratified, multistage, probability sampling method is used to select households that would represent the entire Korean population aged 80 years and younger. The KNHANES VI-1 and -2 surveys separately investigated 11,520 households each and included 7550 and 7380 individuals, respectively. From this pool of participants, individuals aged 60 years or older were included in our current study if they had available data on grip strength, bwt, height, waist circumference, blood pressure, fasting plasma glucose, high-density lipoprotein cholesterol, triglycerides, EuroQoL Five-dimension (EQ-5D) questionnaire, smoking, alcohol consumption, and household income.

To review changes in grip strength, grip strength/bwt, or grip strength/BMI according to age, participants in KNHANES VI-1,2 aged 20 years or older with available grip strength and weight data were analyzed. A total of 4507 men and 5706 women were included in this analysis. This study was approved by the institutional review board of Asan medical center (institutional review board approval no. 2017–1358). Written informed consents were obtained from the subjects.

### Grip strength

Grip strength was measured using a digital grip dynamometer (TKK 5401; Takei Scientific Instruments Co., Ltd., Tokyo, Japan). The dynamometer was adjusted such that the participant could hold the handle comfortably with the palmar eminences and intermediate phalanges. Grip strength was measured three times in each hand with a 1-min rest interval. To be consistent with the recommendations of international working groups on sarcopenia, the highest score of the six measurements was adopted. Medical and physical disabilities that could influence grip strength measurement were assessed by inspection and through a questionnaire. The measurements of any participant with disabilities were omitted. For further analysis, the participants were grouped into quintiles according to grip strength, grip strength/bwt, and grip strength/BMI. Participants with poor grip strength measures were assigned to the lowest quintile group.

### Metabolic syndrome

The definition of metabolic syndrome was based on the 2001 National Cholesterol Education Program-Third Adult Treatment Panel [21]. The cutoff values for waist circumference were adjusted for use in Koreans [22], and the high glucose criterion established in 2003 by the American Diabetes Association was adopted [23]. Metabolic syndrome was defined as the presence of three or more of the following five components: 1) waist circumference ≥ 90 cm in men or ≥ 85 cm in women, 2) triglycerides ≥150 mg/dL, 3) high-density lipoprotein cholesterol < 40 mg/dL in men or < 50 mg/dL in women, 4) blood pressure ≥ 130/85 mmHg or use of antihypertensive medication, and 5) fasting plasma glucose ≥ 100 mg/dL or use of pharmacologic treatment for diabetes mellitus.

### Quality of life

The EQ-5D questionnaire was used to assess QoL. This tool was developed by the EuroQol Group to measure of health-related QoL, and comprises a descriptive section and a valuation section. Only the descriptive section was utilized in KNHANES VI. This part of the survey includes mobility, self-care, usual activities, pain/discomfort, and anxiety/depression, and each dimension is assessed using a three-point Likert scale (i.e., no limitation, some limitation, or extreme limitation). The scores of the five dimensions were converted into a summary index number [24]. Poor QoL was defined by scores in the lowest quintile in the EQ-5D index.

### Anthropologic measurements

Waist circumferences were measured using an ergonomic circumference measuring tape (Seca 201; GmbH & Co. KG, Hamburg, Germany). At the mid-axillary line in the standing position, the inferior ridge of the lowest rib and iliac crest was palpated and the point bisecting the two landmarks was marked. The circumference was measured at this level. The bwt was measured using a portable digital scale (GL-6000-20; Caskorea, Seoul, Korea). The participants were asked to change into an examination gown, stand on the equalized scale, and inhale and then hold their breath. The reading was rounded off to one decimal place after the numbers stabilized. Height was measured using a dedicated height-measuring device (Seca 225; GmbH & Co. KG, Hamburg, Germany). The participants stood straight with bare feet on the horizontal plate with their heel, buttocks, back, and occiput touching the vertical bar. Measurements were done with the participant looking forward and in an inhaled state.

### Environmental factors

The participants were divided into four age groups with 5-year intervals with the exception of the eldest group, which comprised individuals aged 75–80 years. Alcohol consumption, smoking, and household income were assessed using a questionnaire. According to smoking status, the participants were categorized as “non- or ex-smoker” or “current smoker.” With respect to alcohol consumption, the participants were divided into “drinking less than twice a week” and “drinking twice a week or more.” Household income was coded as an ordinal variable according to quartile group.

### Statistical analysis

Independent t-tests for continuous variables and chi-square tests for categorical variables were used to compare the characteristics between sexes. Univariate and multivariate logistic regression analyses were performed to investigate the association of poor grip strength, poor grip strength/bwt, or poor grip strength/BMI with metabolic syndrome or poor QoL. The participants were divided into quintile groups according to grip strength, grip strength/bwt, and grip strength/BMI for each sex and designated Qn (n being a number from 1 to 5), with larger n values indicating poorer strength measures. To examine the dose relationship, logistic regression analyses using quintile groups with the grip strength, grip strength/bwt, or grip strength/BMI of Q1 as a reference were performed. Model 1 was adjusted by age group and model 2 was additionally adjusted by environmental factors such as smoking, alcohol consumption, and household income. Accordingly, odds ratios (ORs) and 95% confidence intervals (CIs) were calculated. The participants’ data were multiplied by the sampling weight to represent the Korean population. Statistical analyses were conducted using SAS version 9.4 (SAS Institute, Cary, NC). Grip strength, grip strength/bwt, and grip strength/BMI data were plotted by age group classified using 5-year increments from age 20 to 80 years.

## Results

**Fig 1:**
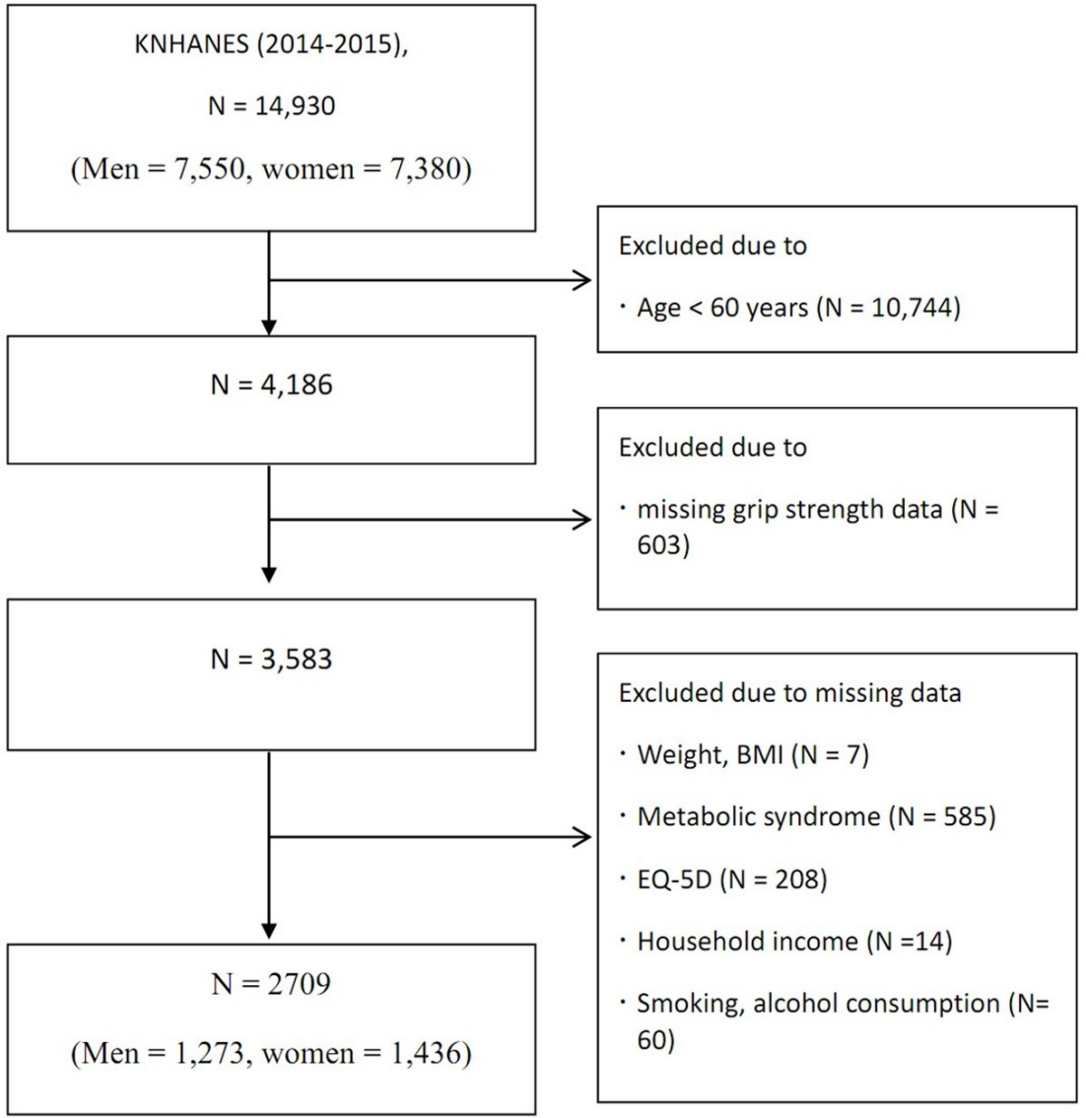
Flow diagram of study participants. KNHANES, Korean National Health and Nutrition Examination Survey; BMI, body mass index; EQ-5D, EuroQol Five-dimension.

Among the whole population, 10,744 participants were aged < 60 years. An additional 1477 persons were excluded owing to the lack of required data, and a total of 2709 participants (1273 men and 1436 women) were examined in our present study (Fig 1). All components of metabolic syndrome differed by sex. A high fasting plasma glucose or triglyceride level was more prevalent in men, whereas women more commonly met the criteria related to blood pressure, high-density lipoprotein cholesterol, or waist circumference. Overall, a higher proportion of women had metabolic syndrome in our present series (Table 1). Environmental factors also differed by sex.

**Table 1:** Basic characteristics of men and women aged 60 years or older in the Korean National Health and Nutrition Examination Survey 2014–2015 (N = 2709).

Characteristics			Men (n = 1273)			Women (n = 1436)			p-Value
Age (years)			68.47	±	0.217	68.74	±	0.197	0.279
Height (cm)			165.76	±	0.177	152.79	±	0.186	0.000
Body weight (kg)			65.82	±	0.286	56.98	±	0.255	0.000
BMI (kg/m^2^)			23.92	±	0.091	24.40	±	0.095	0.000
WC (cm)			86.42	±	0.269	83.46	±	0.329	0.000
FPG (mg/dL)			108.43	±	0.807	105.47	±	0.839	0.011
Systolic BP (mmHg)			125.39	±	0.570	127.80	±	0.526	0.001
Diastolic BP (mmHg)			73.68	±	0.360	73.71	±	0.301	0.950
HDL cholesterol (mg/dL)			46.56	±	0.363	50.12	±	0.367	0.000
Triglyceride (mg/dL)			149.30	±	3.212	133.44	±	2.502	0.000
Grip strength (kg)			36.73	±	0.254	22.98	±	0.158	0.000
Grip strength/body weight			0.56	±	0.004	0.41	±	0.003	0.000
Grip strength/BMI (m^2^)			1.55	±	0.011	0.96	±	0.007	0.000
EQ-5D index			0.93	±	0.004	0.88	±	0.005	0.000
Number of metabolic syndrome components			2.21	±	0.044	2.67	±	0.044	0.000
Metabolic syndrome			512	(40.6)		795	(55.4)		0.000
Components of metabolic syndrome									
	FPG ≥ 100 mg/dL or use of pharmacologic treatment		729	(57.2)		673	(48.8)		0.001
	BP ≥ 130/85 mmHg or use of antihypertensive		831	(62.9)		974	(67.9)		0.022
	HDL < 40 mg/dL for men, < 50 mg/dL for women		380	(30.9)		786	(53.9)		0.000
	Triglyceride ≥ 150 mg/dL		434	(36.0)		453	(31.1)		0.021
	WC ≥ 90 cm for men, ≥ 85 cm for women		423	(33.6)		942	(65.4)		0.000
Environmental factors									
	Household income	Q1	219	(17.2)		198	(13.8)		0.000
		Q2	264	(20.7)		260	(18.1)		
		Q3	401	(31.5)		402	(28.0)		
		Q4	389	(30.6)		576	(40.1)		
	Alcohol consumption ≥ 2/wk		458	(37.2)		78	(5.6)		0.000
	Current smoker		299	(25.0)		37	(2.7)		0.000

Values are presented as mean ± standard error or as number (%). p-Values were obtained using the independent t-test or chi-square test. BMI, body mass index; WC, waist circumference; FPG, fasting plasma glucose; BP, blood pressure; HDL, high-density lipoprotein; EQ-5D, EuroQol Five-dimension; Qn, n^th^ quintile group. The quintile groups are numbered in descending order.

**Fig 2:**
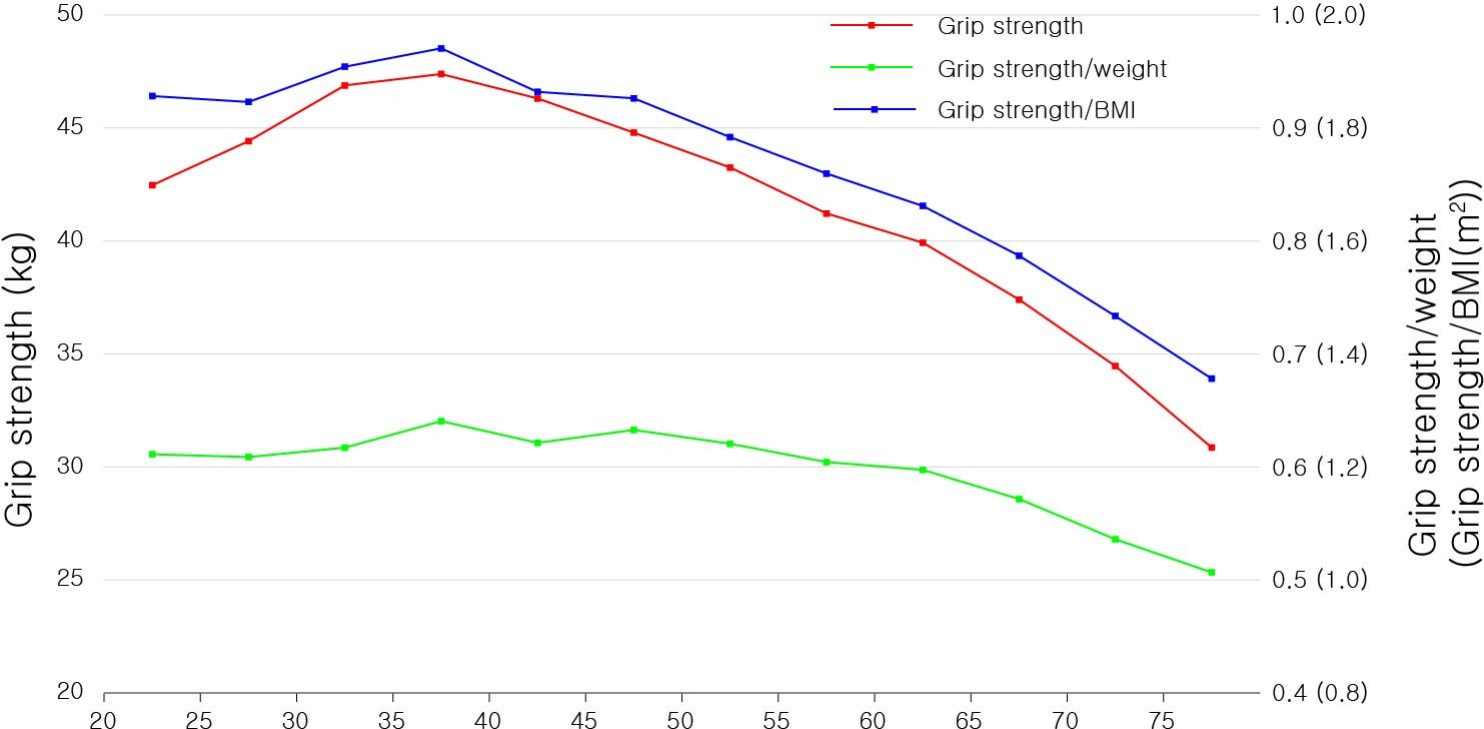
Grip strength, grip strength/body weight, and grip strength/BMI by age in men. BMI, body mass index.

**Fig 3:**
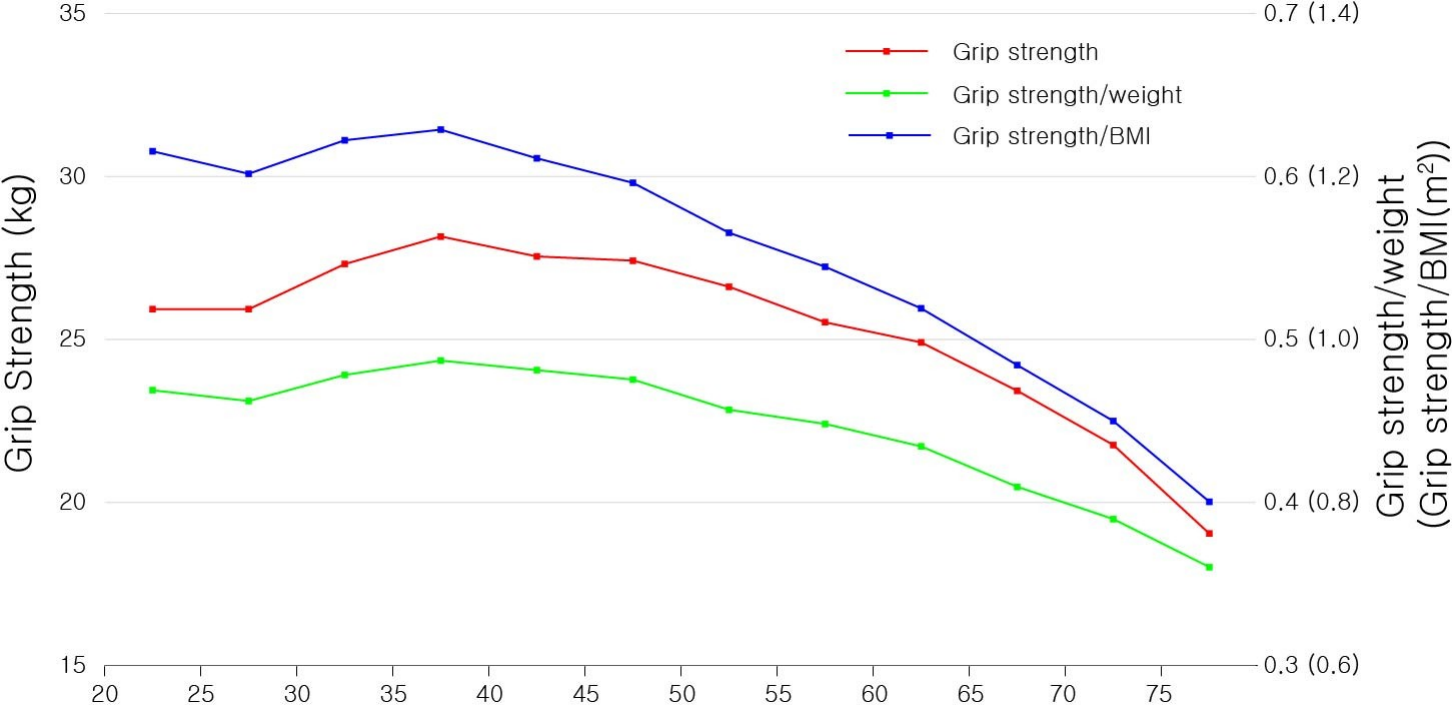
Grip strength, grip strength/body weight, and grip strength/BMI by age in women. BMI, body mass index.

In male participants in our current series, all grip strength measures were the highest in the 35–40 years age group and gradually decreased in the older age groups, with the exception of a slight dip in the 40–45 years age group in normalized grip strength measures, and were slightly aggravated in the gradient beyond age 60–65 years (Fig 2). In women, the grip strength measures were the highest in the 35–40 years age group and steadily decreased in the older age groups with gradient steepening starting from age 45–50 years (Fig 3).

Poor grip strength was not found to correlate with metabolic syndrome development in either sex. However, poor normalized grip strength measures were positively correlated with metabolic syndrome. This tendency persisted even after adjusting for age and environmental factors such as household income, smoking status, and alcohol consumption. The OR (CI) of poor grip strength/bwt and poor grip strength/BMI for metabolic syndrome development was 4.194 (2.985–5.892) and 3.313 (2.243–4.687), respectively, in men and 1.845 (1.283–2.655) and 1.624 (1.124–2.347), respectively, in women in model 2 (Table 2).

**Table 2:** Odds ratios of poor grip strength and poor normalized grip strength for metabolic syndrome.

		Univariate analysis		Model 1		Model 2	
		OR	95% CI	OR	95% CI	OR	95% CI
Men (n = 1273)							
	Grip strength	1.016	0.724–1.428	1.137	0.801–1.612	1.131	0.796–1.608
	Grip strength/bwt	3.659	2.604–5.141	4.198	2.991–5.892	4.194	2.985–5.892
	Grip strength/BMI	2.799	1.989–3.939	3.299	2.344–4.643	3.313	2.342–4.687
Women (n = 1436)							
	Grip strength	1.364	0.973–1.912	1.117	0.787–1.587	1.083	0.762–1.539
	Grip strength/bwt	2.112	1.479–3.016	1.848	1.289–2.650	1.845	1.283–2.655
	Grip strength/BMI	1.906	1.325–2.742	1.639	1.137–2.365	1.624	1.124–2.347

The odds of developing metabolic syndrome in the lowest quintile group relative to the rest were analyzed using logistic regression. OR, odds ratio; CI, confidence interval; bwt, body weight; BMI, body mass index. Model 1 was adjusted by age group. Model 2 was adjusted by age group and environmental factors such as household income, smoking status, and alcohol consumption.

Unlike metabolic syndrome, QoL was correlated with all poor grip strength measures. In model 2, the ORs (CIs) of poor grip strength, poor grip strength/bwt, and poor grip strength/BMI for low QoL were 2.006 (1.314–3.062), 2.015 (1.338–3.035), and 2.119 (1.383–3.245), respectively, in men and 1.612 (1.033–2.515), 2.063 (1.383–3.078), and 2.002 (1.323–3.031), respectively, in women (Table 3).

**Table 3:** Odds ratios of poor grip strength and poor normalized grip strength for poor quality of life.

		Univariate analysis		Model 1		Model 2	
		OR	95% CI	OR	95% CI	OR	95% CI
Men (n = 1273)							
	Grip strength	2.470	1.656–3.686	2.121	1.397–3.220	2.006	1.314–3.062
	Grip strength/bwt	2.311	1.526–3.500	2.095	1.390–3.156	2.015	1.338–3.035
	Grip strength/BMI	2.529	1.651–3.874	2.240	1.462–3.431	2.119	1.383–3.245
Women (n = 1436)							
	Grip strength	2.523	1.694–3.758	1.775	1.147–2.748	1.612	1.033–2.515
	Grip strength/bwt	2.665	1.825–3.891	2.043	1.390–3.003	2.063	1.383–3.078
	Grip strength/BMI	2.748	1.872–4.033	2.068	1.387–3.085	2.002	1.323–3.031

The odds of having a poor quality of life in the lowest quintile group relative to all other groups were analyzed using logistic regression. Poor quality of life was defined as having scores in the lowest quintile in the EuroQol Five-dimension questionnaire. OR, odds ratio; CI, confidence interval; bwt, body weight; BMI, body mass index. Model 1 was adjusted by age group. Model 2 was adjusted by age group and environmental factors such as household income, smoking status, and alcohol consumption.

The mean values of grip strength, grip strength/bwt, and grip strength/BMI by quintile groups are provided in S1 Table. Metabolic syndrome differed only by quintile group divided by normalized grip strength measures. In model 2, the ORs (CIs) of Q2-Q5 were 2.576 (1.653–4.014), 3.014 (1.982–4.583), 4.848 (3.068–7.659), and 11.261 (7.121–17.810) for grip strength/bwt and 2.877 (1.818–4.551), 2.613 (1.682–4.060), 4.054 (2.606–6.307), and 8.601 (5.365–13.788) for grip strength/BMI, which indicate a dose-response relationship. A similar tendency was observed in women. Only normalized grip strength, but not grip strength alone, was related to metabolic syndrome in all analysis models. However, the ORs were attenuated compared with those in men. The ORs (CIs) for metabolic syndrome in Q2-Q5 were 2.199 (1.502–3.220), 2.822 (1.909–4.169), 2.947 (2.011–4.317), and 3.822 (2.508–5.826) for grip strength/bwt and 1.491 (1.034–2.149), 2.646 (1.791–3.907), 2.377 (1.625–3.478), and 2.899 (1.872–4.489) for grip strength/BMI (Table 4).

**Table 4:** Odds ratios of each quintile of grip strength and normalized grip strength for metabolic syndrome.

			Univariate analysis		Model 1		Model 2	
			OR	95% CI	OR	95% CI	OR	95% CI
Men (n = 1273)								
	Grip strength							
		Q1	reference		reference		reference	
		Q2	0.912	0.616–1.350	0.915	0.618–1.355	0.928	0.625–1.377
		Q3	0.754	0.517–1.099	0.782	0.532–1.149	0.778	0.530–1.141
		Q4	0.849	0.554–1.303	0.915	0.584–1.433	0.915	0.583–1.437
		Q5	0.892	0.587–1.355	1.010	0.652–1.563	1.005	0.647–1.561
	Grip strength/bwt							
		Q1	reference		reference		reference	
		Q2	2.432	1.545–3.828	2.493	1.584–3.923	2.576	1.653–4.014
		Q3	2.619	1.725–3.976	2.945	1.93–4.494	3.014	1.982–4.583
		Q4	3.901	2.490–6.111	4.743	2.985–7.539	4.848	3.068–7.659
		Q5	8.272	5.267–12.992	10.992	6.906–17.497	11.261	7.121–17.810
	Grip strength/BMI							
		Q1	reference		reference		reference	
		Q2	2.738	1.731–4.330	2.911	1.832–4.627	2.877	1.818–4.551
		Q3	2.290	1.491–3.520	2.599	1.666–4.055	2.613	1.682–4.060
		Q4	3.203	2.074–4.946	4.049	2.592–6.324	4.054	2.606–6.307
		Q5	6.046	3.841–9.516	8.538	5.327–13.682	8.601	5.365–13.788
Women (n = 1436)								
	Grip strength							
		Q1	reference		reference		reference	
		Q2	1.057	0.756–1.479	0.926	0.655–1.309	0.944	0.669–1.333
		Q3	1.197	0.833–1.721	0.971	0.661–1.427	0.978	0.664–1.439
		Q4	0.928	0.643–1.341	0.696	0.469–1.032	0.694	0.468–1.030
		Q5	1.424	0.970–2.091	0.986	0.650–1.497	0.962	0.632–1.464
	Grip strength/bwt							
		Q1	reference		reference		reference	
		Q2	2.285	1.563–3.341	2.213	1.510–3.243	2.199	1.502–3.220
		Q3	3.009	2.047–4.424	2.827	1.914–4.174	2.822	1.909–4.169
		Q4	3.301	2.271–4.798	2.994	2.051–4.371	2.947	2.011–4.317
		Q5	4.466	2.942–6.777	3.854	2.534–5.861	3.822	2.508–5.826
	Grip strength/BMI							
		Q1	reference		reference		reference	
		Q2	1.615	1.129–2.311	1.511	1.046–2.181	1.491	1.034–2.149
		Q3	2.960	2.010–4.357	2.694	1.819–3.990	2.646	1.791–3.907
		Q4	2.770	1.928–3.978	2.432	1.665–3.553	2.377	1.625–3.478
		Q5	3.481	2.276–5.325	2.966	1.914–4.598	2.899	1.872–4.489

The odds of developing metabolic syndrome in the respective quintile group relative to the first quintile group were analyzed using logistic regression. OR, odds ratio; CI, confidence interval; bwt, body weight; BMI, body mass index; Qn, n^th^ quintile group. The quintile groups are numbered in descending order. Model 1 was adjusted for age. Model 2 was adjusted for age and environmental factors such as household income, smoking status, and alcohol consumption.

Men in Q5 divided by either measure had higher odds to have poor QoL. In model 2, the ORs (CIs) of Q5 by grip strength, grip strength/bwt, and grip strength/BMI were 2.541 (1.348–4.791), 2.182 (1.240–3.840), and 3.049 (1.659–5.602), respectively. Additionally, in women, the Q4 group also had higher odds to have poor QoL and all quintile groups divided by grip strength/BMI had significant ORs with an escalading pattern from Q2 to Q5. In model 2, the ORs (CIs) of Q2-Q5 divided by grip strength/BMI were 2.316 (1.181–4.542), 2.641 (1.270–5.494), 3.716 (1.973–6.999), and 4.991 (2.474–10.065) (Table 5).

**Table 5:** Odds ratios of each quintile of grip strength and normalized grip strength for poor quality of life.

			Univariate analysis		Model 1		Model 2	
			OR	95% CI	OR	95% CI	OR	95% CI
Men (n = 1273)								
	Grip strength							
		Q1	reference		reference		reference	
		Q2	0.824	0.418–1.627	0.817	0.414–1.612	0.803	0.403–1.597
		Q3	1.511	0.825–2.767	1.442	0.754–2.758	1.379	0.718–2.646
		Q4	1.984	1.051–3.744	1.834	0.930–3.617	1.652	0.834–3.273
		Q5	3.167	1.794–5.592	2.818	1.510–5.259	2.541	1.348–4.791
	Grip strength/bwt							
		Q1	reference		reference		reference	
		Q2	1.059	0.557–2.013	1.066	0.559–2.033	1.074	0.566–2.037
		Q3	1.187	0.646–2.178	1.101	0.593–2.046	1.135	0.609–2.117
		Q4	1.196	0.639–2.240	1.083	0.577–2.033	1.112	0.591–2.092
		Q5	2.554	1.440–4.530	2.229	1.258–3.949	2.182	1.240–3.840
	Grip strength/BMI							
		Q1	reference		reference		reference	
		Q2	1.472	0.831–2.605	1.445	0.810–2.580	1.441	0.803–2.586
		Q3	1.741	0.947–3.200	1.635	0.874–3.057	1.580	0.849–2.941
		Q4	1.844	1.012–3.361	1.660	0.894–3.083	1.630	0.868–3.063
		Q5	3.784	2.119–6.757	3.266	1.787–5.971	3.049	1.659–5.602
Women (n = 1436)								
	Grip strength							
		Q1	reference		reference		reference	
		Q2	1.167	0.561–2.427	0.995	0.471–2.104	1.030	0.494–2.148
		Q3	2.227	1.207–4.111	1.731	0.922–3.249	1.785	0.949–3.360
		Q4	3.372	1.873–6.061	2.393	1.306–4.384	2.540	1.382–4.668
		Q5	4.508	2.415–8.416	2.784	1.411–5.492	2.602	1.327–5.099
	Grip strength/bwt							
		Q1	reference		reference		reference	
		Q2	2.199	1.152–4.196	2.085	1.096–3.968	2.153	1.143–4.056
		Q3	1.914	1.010–3.626	1.687	0.894–3.185	1.668	0.880–3.159
		Q4	3.218	1.770–5.851	2.571	1.396–4.737	2.531	1.373–4.663
		Q5	5.316	2.864–9.869	3.761	1.999–7.078	3.789	2.008–7.150
	Grip strength/BMI							
		Q1	reference		reference		reference	
		Q2	2.680	1.353–5.309	2.279	1.148–4.524	2.316	1.181–4.542
		Q3	3.399	1.686–6.850	2.678	1.275–5.627	2.641	1.270–5.494
		Q4	5.332	2.925–9.718	3.806	2.013–7.198	3.716	1.973–6.999
		Q5	7.855	4.054–15.222	5.210	2.577–10.000	4.991	2.474–10.065

The odds of developing metabolic syndrome in the respective quintile group relative to the first quintile group were analyzed using logistic regression. OR, odds ratio; CI, confidence interval; bwt, body weight; BMI, body mass index; Qn, n^th^ quintile group. The quintile groups were numbered in descending order. Model 1 was adjusted for age. Model 2 was adjusted for age and environmental factors such as household income, smoking status and alcohol consumption.

In the QoL analyses stratified by quintile group divided by either grip strength or normalized grip strength, Q5 cases were most likely to have poor QoL than Q1 cases in both sexes. Among the female participants, Q4 was also more likely to have poor QoL than Q1 in terms of grip strength and normalized grip strength. Interestingly, the ORs for poor QoL were larger in Q5 defined by grip strength/BMI than those defined by grip strength/bwt. In women, all quintile groups divided by grip strength/BMI were more likely to have poor QoL than Q1 with consistently increasing ORs from Q2 to Q5.

## Discussion

In this study, we analyzed the relationship between grip strength, grip strength/bwt, or grip strength/BMI and metabolic syndrome or QoL using the data of 1273 men and 1436 women aged 60–80 years. Poor grip strength was not associated with metabolic syndrome, whereas poor normalized grip strength measures were associated with metabolic syndrome in both sexes with a dense dose-response relationship. All grip strength measures were associated with poor QoL. Between normalized grip strength measures, grip strength divided by bwt was better associated with metabolic syndrome whereas grip strength divided by BMI seemed more related to QoL.

Sarcopenia is defined by low muscle mass, weak muscular strength, and poor physical performance. Although sarcopenia is closely associated with many health conditions and major clinical outcomes, it is impractical to assess all dimensions of sarcopenia in daily clinical practice because the assessment of muscle mass requires specialized equipment and the evaluation of physical performance takes time and space. On the other hand, grip strength measurement is simple and thus convenient to implement in the physicians’ office. However, simple grip strength does not sufficiently represent all components of the muscle, which raises the need to modify this measure to encompass more extensive aspects of sarcopenia.

The mass and strength of a muscle increase when it continuously meets sufficient resistance. Thus, if the amount and extent of physical activity of a given group of participants are similar, the muscle strength will be proportional to the bwt, as this will act as a load. Numerous studies to date have reported that muscle strength positively correlates with bwt, and studies that use muscle strength as an outcome variable usually adopt a method of normalizing this variable by body mass [25]. However, the definition of sarcopenia includes only simple muscle strength criteria. Thus, we investigated the relationship between muscle strength and muscle strength divided by either bwt or BMI and two major correlates of sarcopenia, namely metabolic syndrome and QoL. Our results indicated that differences in the relationship between the two strength measures diverged with respect to these correlates.

Poor grip strength was not found to correlate with metabolic syndrome, whereas poor normalized grip strength correlated with metabolic syndrome with a dense dose-response relationship in both sexes. As obesity is closely related to metabolic syndrome, it is intuitive that a measure with a reciprocal of bwt would be negatively associated with metabolic syndrome. Comparing bwt and BMI by themselves, BMI is better correlated with metabolic syndrome than bwt and major surveys adopt BMI to represent body fatness [26]. However, in the current study results, grip strength normalized by BMI was less associated with metabolic syndrome compared with grip strength normalized by bwt. This implies that the correlation between normalized grip strength and metabolic syndrome is not entirely due to the incorporation of body mass into the measure. Instead, there is a link between strength and metabolic syndrome that is not shown in the regression analysis between the two factors.

Grip strength is correlated with muscle mass [27], and muscle acts as an endocrine organ that protects against metabolic syndrome [3]. Therefore, the lack of a significant correlation between grip strength itself and metabolic syndrome in the current analyses was unexpected. In the general population, muscle mass and fat mass are proportional to each other [28]. Thus, grip strength will also be correlated with fat mass, which consequently balances out the negative association with metabolic syndrome. Another explanation is that grip strength is determined by the anterior forearm muscles, which account for a small proportion of the whole-body muscle and might not effectively represent whole-body muscle mass [29].

Previous studies that investigated the relationship between muscle strength and metabolic syndrome were not in agreement, as some reports indicated no association [30,31] whereas others described an inverse relationship [6,16,32]. Normalizing muscle strength by lean mass is theoretically correct with respect to representing muscular function. However, bwt seems to be superior in predicting metabolic syndrome. Atlantis et al. compared the prevalence of metabolic syndrome in quartiles grouped by grip strength/arm lean mass, with the best quartile as a reference in men. The ORs of the first and second worst quartiles were 2.15 and 1.91, respectively, in that study [6]. These values are considerably smaller than those in our current study, which is likely because of differences in normalization. Normalizing grip strength with bwt would be superior than normalizing with lean mass in terms of predicting metabolic syndrome because fat plays an important role in the development of this disorder.

There have been many published reports on the relationship between muscle strength and QoL. Some studies did not find any significant relationship between the two factors [7,18]; however, previous large population-based studies reported significant associations [33,34]. Muscle strength seems to be more correlated with QoL in women than in men [34] and in patients than in healthy participants [35]. All studies that reported muscle strength in both sexes or in both healthy participants and patients reported lower strength in women and in patients [7,18,36,37]. This implies that muscle strength has a greater influence on QoL in weaker persons who may not be able to perform daily life activities.

In the current analyses, grip strength was found to be associated with QoL in both sexes and normalizing grip strength by body mass did not notably alter the relationship in general. Given that hampered mobility in old adults is a major cause of poor QoL [38], we expected that the normalized grip strength measures would have a stronger correlation with QoL than simple grip strength. There is a ceiling in the positive effect of muscular strength on mobility [39], and the muscular strength required for daily activities might not be large enough to enable grip strength to affect the QoL. The correlation between grip strength and QoL could be due to aspects of muscle other than strength. If strength is not the link between muscle and QoL, dividing grip strength by body mass would not affect its capability to predict QoL. On the other hand, as shown in the quintile groups with weaker grip strength, normalizing grip strength by body mass would enhance its relationship with QoL in persons whose strength is poor enough to limit daily activities.

Participants in the poorer quintile groups classified by grip strength/BMI seemed to have higher odds to have poor QoL than those classified by grip strength/bwt. The reason why grip strength/BMI is better correlated with QoL may be the incorporation of height in the measure. Height loss in the elderly is related to health conditions [40] such as degenerative kyphosis and vertebral fractures. These conditions can cause bodily pain and problems in locomotive function, ultimately hampering the QoL.

The grip strength in old adult participants reported in our current study is similar to that in other reports based on nationwide population-based data from East Asian countries [41,42]. Our current results for grip strength were also similar to those reported in studies from Western countries [43,44] or lower than those in others [45,46]. Among the prior studies that reported higher grip strength, one also reported significantly higher bwt [45] than that in our present analysis. Although statistical testing was not possible, when dividing the mean grip strength by mean bwt, both studies showed similar values. This suggests that the strength difference is largely explained by differences in body mass. Grip strength peaked in the group of participants in their late 30s in both sexes, although more prominently in men than in women, and decreased thereafter. This pattern was found worldwide with some divergence in the peak age [46].

The main strength of our current study is that it is the first report to articulate the difference in the relationships of muscle strength and normalized muscle strength to metabolic syndrome and QoL. It was also based on nationwide population-based data. However, there were also some notable limitations in our present analyses. First, no causal relationship could be derived from our current findings owing to the cross-sectional study design. Second, we did not analyze limited physical performance and mortality, which also are important consequences of sarcopenia; thus, we could not conclude which measure better reflects sarcopenia in general. Third, we adopted grip strength as a measure; however, grip strength might not represent the whole-body muscle mass or physical performance. Thus, it would not be the best muscular measurement to predict metabolic syndrome or QoL. Fourth, normalization by dividing grip strength with bwt does not accurately reflect muscular function. However, its applicability in clinical settings is a major strength of this measure. Hence, a ready assessment of strength by evaluating grip strength and normalization using a simple bwt value was adopted in our analysis.

## Conclusion

Grip strength is not related to metabolic syndrome; however, normalizing grip strength by dividing it by bwt or BMI gives it predictive value, with normalization with bwt being superior to normalization with BMI with respect to this disorder. Grip strength and normalized grip strength show a similar association with QoL. Hence, when discussing the metabolic aspects of the muscle in clinical research, bwt should be incorporated into the normalization of this strength measure. Further studies that assess physical performance or mortality would help in developing a strength measure that is a better indicator of sarcopenia.

## Supporting information

S1 Table Values of grip strength, grip strength/body weight, and grip strength/body mass index in each quintile group. (DOCX)
