# Supplementary material for: Comparison between grip strength and grip strength divided by body weight in their relationship with metabolic syndrome and quality of life in the elderly
Source: PLoS One. 2019 Sep 6;14(9):e0222040. doi: 10.1371/journal.pone.0222040 (PMC6730916; doi:10.1371/journal.pone.0222040)
Supplement: S1 Table — (DOCX) [file pone.0222040.s001.docx]

**S1 Table.** Values of grip strength, grip strength/body weight, and grip strength/body mass index in each quintile group

|  |  | Grip strength (kg) | | |  | Grip strength/bwt | | |  | Grip strength/BMI (m^2^) | | |
| --- | --- | --- | --- | --- | --- | --- | --- | --- | --- | --- | --- | --- |
| Men | |  |  |  |  |  |  |  |  |  |  |  |
|  | Q1 | 45.50 | ± | 0.177 |  | 0.70 | ± | 0.003 |  | 1.96 | ± | 0.009 |
|  | Q2 | 39.47 | ± | 0.061 |  | 0.61 | ± | 0.001 |  | 1.68 | ± | 0.003 |
|  | Q3 | 36.03 | ± | 0.059 |  | 0.56 | ± | 0.001 |  | 1.52 | ± | 0.002 |
|  | Q4 | 32.18 | ± | 0.066 |  | 0.50 | ± | 0.001 |  | 1.37 | ± | 0.003 |
|  | Q5 | 25.81 | ± | 0.216 |  | 0.40 | ± | 0.003 |  | 1.08 | ± | 0.009 |
|  |  |  |  |  |  |  |  |  |  |  |  |  |
| Women | |  |  |  |  |  |  |  |  |  |  |  |
|  | Q1 | 28.65 | ± | 0.108 |  | 0.52 | ± | 0.002 |  | 1.23 | ± | 0.006 |
|  | Q2 | 24.85 | ± | 0.041 |  | 0.44 | ± | 0.001 |  | 1.04 | ± | 0.002 |
|  | Q3 | 22.33 | ± | 0.037 |  | 0.40 | ± | 0.001 |  | 0.91 | ± | 0.002 |
|  | Q4 | 19.62 | ± | 0.045 |  | 0.35 | ± | 0.001 |  | 0.80 | ± | 0.002 |
|  | Q5 | 15.05 | ± | 0.120 |  | 0.27 | ± | 0.002 |  | 0.61 | ± | 0.005 |

Values are presented as mean ± standard error. bwt, body weight; BMI, body mass index
